# Skill in discrete keying sequences is execution rate specific

**DOI:** 10.1007/s00426-017-0967-2

**Published:** 2018-01-03

**Authors:** Willem B. Verwey, Wouter J. Dronkers

**Affiliations:** 1 Department of Cognitive Psychology and Ergonomics, University of Twente, PO Box 217, 7500 AE Enschede, The Netherlands; 2 Human Performance Laboratories, Department of Health and Kinesiology, Texas A&M University, College Station, TX USA

## Abstract

The present study tested the hypothesis that in motor sequences, the interval between successive movements is critical for the type of representation that develops. Participants practiced two 7-key sequences in the context of a discrete sequence production (DSP) task. The 0-RSI group practiced these sequences with response stimulus intervals (RSIs) of 0, which is typical for the DSP task, while the long-RSI group practiced the same sequences with unpredictable RSIs between 500 and 2000 ms. The ensuing test phase examined performance of these familiar and of unfamiliar sequences for both groups under both RSI regimes. The results support our hypothesis that the motor chunks that 0-RSI participants developed could not be used with long RSIs, whereas the long-RSI participants developed sequence representations that cannot be used with 0 RSIs. A new, computerized, sequence awareness task showed that long-RSI participants had limited sequence knowledge. The sequencing skill developed by long-RSI participants can, therefore, not have been based on explicit knowledge.

## Introduction

Motor skills play a crucial role in our lives. Skilled performance of tasks like car driving, playing video games, and playing soccer is possible only because people can develop behavioral “building blocks” that consist of fixed movement patterns to perform automated subtasks like shifting gears, dealing with recurring virtual enemies, and ball dribbling. Evidence for the use of such building blocks has been reported for various real-world tasks, such as typing (Viviani & Laissard, 1996; Yamaguchi, Crump, & Logan, 2012), video games (Thompson, McColeman, Stepanova, & Blair, 2017), and building LEGO walls (Arnold, Wing, & Rotshtein, 2017). These building blocks can be practiced in isolation in the situation that in the eventual task, they are kinematically independent (Fontana, Mazzardo, Furtado Jr, & Gallagher, 2009). When people develop into experts in a particular task, their behavioral building blocks become highly idiosyncratic. This has been found with, for example, flute players (Albrecht, Janssen, Quarz, Newell, & Schöllhorn, 2014) and professional typists (Viviani & Laissard, 1996). Experts can flexibly adjust movement execution if circumstances change (MacKay, 1982), which suggests that they can switch between strategies and building blocks. Another benefit of the proficiency to integrate movements into behavioral building blocks is that this allows the information processing system to deal with limitations in information processing capacity at a central level, so that mental overload is prevented (e.g., Fonollosa, Neftci, & Rabinovich, 2015; Halford, Wilson, & Phillips, 1998; Ramkumar et al., 2016). In practice, motor skills are often learned by the trainee observing and mimicking a human model who demonstrates the goal behavior (Badets & Blandin, 2005; Ellenbuerger, Boutin, Blandin, Shea, & Panzer, 2012; Wolpert, Diedrichsen, & Flanagan, 2011).

To study the characteristics and the development of these behavioral building, blocks in the laboratory researchers use various sequencing tasks. These include, for example, fixed forearm rotation sequences in the flexion–extension (FE) task, and fixed key pressing sequences in the serial reaction time (SRT) task and the discrete sequence production (DSP) task. In these tasks movement order is indicated by successively presented element-specific stimuli to which the participants initially react (for reviews of these three paradigms, see Abrahamse, Jiménez, Verwey, & Clegg, 2010; Abrahamse, Ruitenberg, De Kleine, & Verwey, 2013; Keele, Ivry, Mayr, Hazeltine, & Heuer, 2003; Shea, Kovacs, & Panzer, 2011; Shea, Panzer, & Kennedy, 2016).

In the present study, we focused on the DSP task. In this task, participants are typically guided by two fixed series of 3–7 stimuli, each indicating a key press, so that eventually they develop the skill to rapidly execute two fixed, discrete keying sequences. Below, we derive and test the hypothesis that in the DSP task, the building blocks cannot be used when the motor sequence is executed substantially slower or faster than during practice.

### Multiple sequence representations

Research with the DSP task has provided ample support for the idea that motor sequencing skill is based on representations in memory that reduce and eventually even eliminate the reliance on element-specific stimuli (Abrahamse et al., 2013; Rhodes, Bullock, Verwey, Averbeck, & Page, 2004). This idea is based on findings like individual key presses in a familiar keying sequence becoming so fast that stimulus-based selection of individual responses is unlikely. Execution rate of such familiar sequences appears to decrease only little when element-specific stimuli are no longer displayed, while in contrast, execution rate decreases substantially if only a single element is being altered (Abrahamse et al., 2013; Verwey, 1999, 2010). There is general consensus now that skilled motor behavior is based on a practice-, task-, and age-dependent mixture of various sequence representations (Panzer, Gruetzmacher, Ellenbuerger, & Shea, 2014; Shea et al., 2016; Verwey, Shea, & Wright, 2015; Wiestler, Waters-Metenier, & Diedrichsen, 2014). These ideas have recently been worked out in the cognitive framework for Sequential Motor Behavior (C-SMB; Verwey et al., 2015) which distinguishes between motor chunks, spatial, and verbal central-symbolic sequence representations, and associative sequence representations. These representations may well be based in independent neural systems that are racing to trigger each next movement in the sequence (Verwey, 2003b).

Motor chunks have been argued to involve successions of agonist/antagonist muscle activation patterns (Shea et al., 2011), musculoskeletal forces and dynamics (Krakauer, Ghilardi, & Ghez, 1999), joint angles (Criscimagna-Hemminger, Donchin, Gazzaniga, & Shadmehr, 2003), body postures (Rosenbaum et al., 2009), and/or successive orientations of body segments relative to each other (Lange, Godde, & Braun, 2004). Participants using motor chunks are said to perform movement sequences in the *chunking mode* (Verwey & Abrahamse, 2012).

Spatial representations would differ with respect to their reference frame, which may be relative to some point in the outside world in the case of allocentric representations, or relative to some body part with egocentric representations (Barnhoorn, Döhring, Van Asseldonk, & Verwey, 2016; Liu, Lungu, Waechter, Willingham, & Ashe, 2007; Verwey, Groen, & Wright, 2016; Witt, Ashe, & Willingham, 2008). Verbal representations may be used especially with unfamiliar motor sequences, like when one types for the first few times a verbally learned PIN code or phone number (Fendrich & Arengo, 2004; Fendrich, Healy, & Bourne Jr, 1991). These verbal and spatial representations are said to underlie the so-called central-symbolic execution mode (Verwey et al., 2015). The responsible central-symbolic representations develop more rapidly than motor chunks, but they are associated with lower execution rates than motor chunks because it takes substantial processing to extract the individual movements from these abstract representations (Hikosaka et al., 1999; Verwey et al., 2015). The more abstract a sequence representation the slower execution of the motor sequence that it controls. Yet, this online movement extraction process also makes sequencing skill flexible when the situation changes.

Finally, associations between successive movement-specific representations have been argued to underlie the associative mode. These associations prime the ensuing response at perceptual, central and motor processing levels (Abrahamse et al., 2010). In contrast to the chunking mode, the associative mode still requires stimuli for selecting and executing the individual responses (for a similar distinction between priming and selecting responses, see Kornblum, Hasbroucq, & Osman, 1990). Associative learning is generally considered the primary way of sequence learning in the SRT task (Abrahamse et al., 2010; Keele et al., 2003), and this construct is probably closely related to the construct of statistical learning (Hunt & Aslin, 2001; Perruchet & Pacton, 2006). Associative learning has been argued also to support motor chunk based learning in the DSP task (Verwey & Abrahamse, 2012; Verwey, Abrahamse, Ruitenberg, Jiménez, & De Kleine, 2011).

### Explicit sequence knowledge

Often neglected in motor sequencing studies is the fact that some participants can give a full verbal account of the acquired movement sequence while others cannot, or only to a limited degree. These participants are said to have explicit sequence knowledge (Frensch & Rünger, 2003; Shanks & John, 1994; Willingham, Nissen, & Bullemer, 1989). Explicit sequence knowledge has been defined, for example, as knowledge that can be “reported, reasoned about, and used for voluntary action” (Rünger & Frensch, 2010, p. 128).

The availability of explicit knowledge probably benefits sequence execution in several respects. First, participants have been found to rely more on the chunking mode when they have more explicit knowledge of the sequence (Verwey et al., 2015). The reason is probably that being aware of having this sequence knowledge stimulates participants to use the chunking mode (Dienes & Scott, 2005; Stanley & Krakauer, 2013). Second, explicit sequence knowledge allows flexibility like when a PIN code is typed on a numeric key pad with an unfamiliar layout^1^. Third, explicit sequence knowledge facilitates also the execution of well-practiced sequences. This follows from observations that participants with substantial explicit sequence knowledge are often faster on these sequences. This has been observed in both the SRT task (Curran & Keele, 1993; Mayr, 1996; Rüsseler, Kuhlicke, & Münte, 2003) and the DSP task (Ruitenberg, Abrahamse, De Kleine, & Verwey, 2012; Verwey & Abrahamse, 2012; Verwey, Abrahamse, & De Kleine, 2010; Verwey et al., 2011). This benefit of explicit sequence knowledge seems larger as the sequence is executed at lower execution rates, like with limited practice (Verwey & Wright, 2014), when deviating stimuli are expected (Verwey, 2015; Verwey & Abrahamse, 2012), and when participants are older (Barnhoorn, Van Asseldonk, & Verwey, 2017).

Recently, an analysis across six DSP studies showed that in a paper-and-pencil awareness test, 53% of a total of 168 participants had been able to write down all elements of their 6- or 7-key sequences in the proper order (Verwey et al., 2016). However, when asked 64% of these participants indicated to have reconstructed their sequences in the awareness test by playing them off in their mind or using their fingers on the table top. This suggests that paper-and-pencil tests are influenced by implicit sequence knowledge and over-estimate the amount of explicit sequence knowledge that can be directly retrieved from memory. Nevertheless, 21% of the participants still indicated to have used spatial sequence knowledge when they were writing down their sequences and 15% said they had been using verbal sequence knowledge. This leaves open the possibility that at least some of the participants in DSP studies do use directly accessible explicit spatial and/or verbal sequence representations when filling out the paper-and-pencil test.

Currently, it is not clear how explicit sequence knowledge and the earlier mentioned central-symbolic sequence representations are related to each other. They are based on different tests, but it lies at hand that the explicit sequence knowledge retrieved directly from memory is based on the same central-symbolic sequence representations that are suggested by analyses of sequence performance.

### Effects of sequence execution rate

The integration of successive movements into motor chunks seems based on associations between their memory representations (Abrahamse, Van der Lubbe, Verwey, Szumska, & Jaśkowski, 2012; Brown & Carr, 1989; Frensch & Miner, 1994; MacKay, 1982; Verwey et al., 2015). These associations would develop according to the postulate that “neurons wire together if they fire together” (Hebb, 1949; Lowel & Singer, 1992). For motor sequences, this implies that when the representations of successive movements are activated in close temporal proximity, they gradually become associated and form a motor chunk.

It is generally assumed that the activation of memory units decays over time (Frensch & Miner, 1994; Hommel, 1994; Mueller, Seymour, Kieras, & Meyer, 2003). This has clear ramifications for both learning and execution of motor sequences: It suggests that sequence representations develop more slowly when there is more time between successive movements, and thus that the benefit of practice reduces as the sequence is carried out more slowly. This effect of RSI may be strengthened because the frustration and boredom caused by slow sequence execution may reduce the tendency to prepare oncoming actions and therewith reduce further the co-activation of movement representations (Willingham, Greenberg, & Thomas, 1997).

The effect of execution rate on sequence learning has not yet been investigated in the DSP task, but the sequence learning literature does provide some support for the idea that slower execution reduces learning of keying discrete motor sequences too. In the related SRT task, implicit sequence knowledge developed more slowly when RSIs were longer (Frensch & Miner, 1994; Soetens, Melis, & Notebaert, 2004). Furthermore, the occurrence of long intervals at specific sequential locations seems to induce less binding between sequence elements, which eventually is responsible for a segmentation structure that is still used when executing rates are high (Bower & Winzenz, 1969; Shea et al., 2016; Stadler, 1993; Verwey, Abrahamse, & Jiménez, 2009). Finally, skill in the DSP task with its 0 RSIs did not transfer to the serial reaction time (RT) task with its 200 ms RSIs when familiar DSP segments were inserted in an SRT task sequence (Verwey, 2003b).

Nevertheless, there may also be an advantage of long intervals between successive movements in that they may facilitate the development and use of explicit sequence knowledge. The reason is that the longer inter-element intervals would allow participants to develop, test and apply hypotheses with respect to the order of sequence elements (Frensch & Miner, 1994; Rünger & Frensch, 2008). Indeed, in the SRT task, participants developed more explicit sequence knowledge when RSIs were longer (Cleeremans & Sarrazin, 2007; Destrebecqz & Cleeremans, 2001), and in the DSP task, the impact of existing explicit sequence knowledge was higher with low than with high execution rates (Verwey & Abrahamse, 2012; Verwey & Wright, 2014).

Together, these findings suggest that different representations develop when practicing motor sequences at low and at high execution rates and that these representations can be applied best at the execution rate at which they originally developed (much like the specificity of practice notion with aiming movements, Proteau, 1992).

### The present study

In the present experiment, we had a 0-RSI group practicing two 7-key sequences with 0 RSIs, and a long-RSI group practicing the same sequences with RSIs between 500 and 2000 ms. In the ensuing test phase, both groups performed in the same four test conditions. These included familiar and unfamiliar sequences produced under the 0-RSI and long-RSI regimes that had also been used during practice. We tested the hypothesis that sequencing skill is limited to the execution rate at which this skill had previously developed. Specifically, the 0-RSI practice participants were expected to develop motor chunks that can be used only in the 0-RSI condition, and the long-RSI practice participants were expected to develop sequence representations that can only be used in the long-RSI condition. To examine the contribution of explicit knowledge to sequence execution, we had the participants carry out a new computerized awareness test. This task allowed us to exclude slow responses that might result from reconstructing the sequence on the basis of other types of sequence knowledge. It further involved a spatial test and a verbal test to examine the type of representation. Higher awareness levels in the long-RSI than in the 0-RSI participants could indicate that long-RSI participants develop a sequencing skill based on explicit sequence knowledge.

## Method

### Participants

A total of 24 participants performed in the present experiment (16 males, *M* age = 21, SD = 2.7 years). Fourteen volunteered while ten of them received 5 euro for participation. This study was approved by the ethics committee of the Faculty of Behavioral Sciences of the University of Twente, Enschede, The Netherlands. Prior to the experiment, all participants filled out an informed consent form. The participants were naive with respect to the DSP task. They were randomly assigned to the 0-RSI and long-RSI groups.

### Apparatus

Presentation of stimuli and registration of responses were controlled and registered by E-Prime 2.0 on a Toshiba laptop running under Windows XP. Unnecessary Windows services had been removed to improve response time measurement accuracy. The experiment took place in a quiet, moderately lit room with just a desk and a chair.

### Tasks

The DSP task started off by having the participant rest their left index and middle fingers on the C and V keys, and the right index and middle fingers on the B and N keys. Four 2 × 2 cm black placeholders with a default white filling were presented on a white background on the screen. The stimulus consisted of a green filling of one of these placeholders, which was to be followed by pressing the spatially compatible key. Key release was not registered and could in principle follow depression of the ensuing key. Each participant practiced two 7-key sequences that were selected from a set of four counterbalanced sequences: VCBNCVN, NVCBVNB, BNVCNBC, and CBNVBCV. This counterbalancing involved rotating between the four keys (V→N→B→C→ etc.), so that, across participants, each finger occurred equally often at each sequential position. In addition, across participants, each sequence was used equally often as familiar and as unfamiliar sequence in the test block (see below). Stimuli, responses, and the time between each stimulus and responses are designated by *S, R*, and *T* followed by an index showing their position in the sequence (yielding *S*_1_–*S*_7_, *R*_1_–*R*_7_, and *T*_1_–*T*_7_, respectively).

In Blocks 1 through 7 (i.e., the practice phase), the 0-RSI group practiced with a 0 RSI and the long-RSI group with non-aging RSIs between 500 and 2000 ms. Each practice block included 30 repetitions of each of the two sequences. These sequences were performed in a random order. This yielded 210 practice trials for each sequence. This is somewhat less than half the typical number of about 500 practice trials in the regular DSP studies. Each block involved a 20 s break halfway through, and each block was followed by a 180 s break. During the long breaks, the participants were asked whether they were tired and, if so, starting the next block was delayed. In both RSI groups, pressing the last key of a sequence, or an erroneous one, was followed by the display being entirely erased. After 1000 ms, the placeholders were displayed again for another 1000 ms, after which the first placeholder was filled. At the end of each subblock, the average response time and error rate were displayed. Participants were urged to keep error rate below 6%, but there were not sanctions if it was higher. When a false key was pressed the sequence was broken off, an error message was displayed for 2.5 s and the next sequence commenced after the usual inter-sequence interval of 2 s. The relatively long message display was meant to encourage participants to prevent errors.

After the practice phase, participants performed a computerized awareness task consisting of two tests. While the keyboard was covered, participants clicked on the display with the computer mouse the seven successive elements of each of the two practiced sequences. In the spatial test, these elements were represented by square placeholders that were lined up next to each other, just like in the practice phase. Participants were asked to click the placeholders in the order in which the stimuli had appeared, once for each of the participant’s two sequences. In the verbal test, four square placeholders were positioned in a rhombus shape with the placeholders distributed across the top, left, bottom, and right part of the display. This time, each placeholder contained one of the four letters of the keys the participants had been pressing during practice. The participants were to click the order in which the keys had been pressed during execution of each of the two sequences.

After completing the awareness task, the participants had a break which ended by the experimenter starting the test block (Block 8). It contained 4 subblocks, each including 48 trials, 24 trials with each sequence. These were, again, presented in a random order. Two subblocks involved the same (i.e., familiar) sequences that each participant had just been practicing in the practice phase. The other two subblocks involved two unfamiliar sequences, which were the two sequences of the set of four the participants had not yet carried out. One of the familiar and one of the unfamiliar sequence subblocks involved a 0 RSI. The other two subblocks involved the long and non-aging RSIs, like those used by the long-RSI participants. Prior to each test condition, the participants were informed on the computer display as to the upcoming stimulus display rate and familiarity of the sequences. The order of these four subblocks was counterbalanced across participants.

### Procedure

Upon entering the lab, the participants received a written instruction on the task to be performed. On request, the experimenter provided further explanations. They were explicitly told to take care that no more than 6% of the sequences had an error, and this instruction was repeated in the on-screen instruction. The participants then filled out the informed consent form and carried out the seven practice blocks. At the end of the practice phase, the participants performed the awareness task, followed by the test block. For the 0-RSI group, the experiment took about 1 h and 15 min, for the long RSI group about 2 h and 20 min.

## Results

### Practice phase

**Fig. 1:**
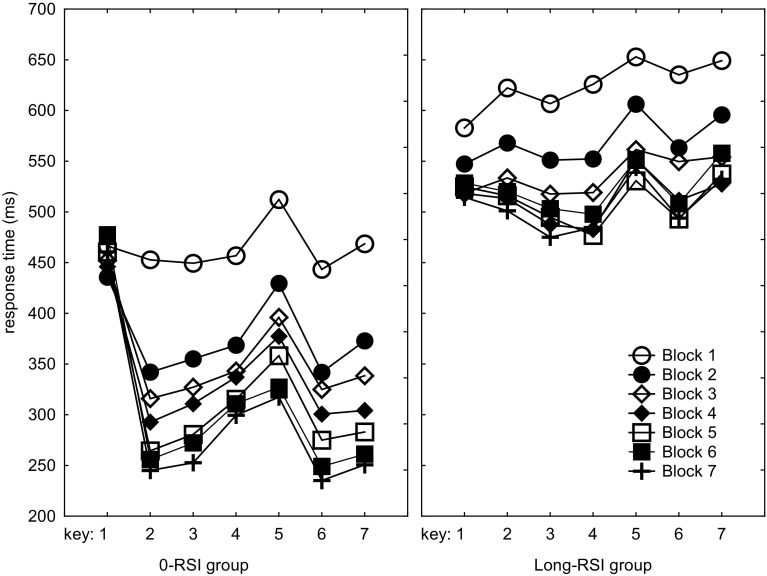
Response times as a function of practice group and key position across the seven practice blocks. In the long-RSI group, RSIs consisted of non-aging intervals between 500 and 2000 ms

Response times in the 7 practice phase blocks were analyzed using a 2 (RSI group: 0-RSI vs. long-RSI) × 7 (Block) × 7 (Key) mixed ANOVA with RSI group as between-subjects variable. It showed the usual effects of Block, *F*(6,132) = 27.5, *p* < 0.001, *η*_*p*_^2^ = 0.56, and Key, *F*(6,132) = 11.4, *p* < 0.001, *η*_*p*_^2^ = 0.34, indicating that RT reduced across successive blocks and differed for the various positions in the sequence (Fig. 1). RSI group also showed a main effect, *F*(1,22) = 12.0, *p* < 0.01, *η*_*p*_^2^ = 0.35, and RSI group interacted with Key, *F*(6,132) = 9.9, *p* < 0.001, *η*_*p*_^2^ = 0.31. This showed that the 0-RSI group generally had shorter RTs than the long-RSI group (351 vs. 541 ms), and that the RTs across serial positions followed different patterns for the two groups. The Block × Key interaction, *F*(36,792) = 9.0, *p* < 0.001, *η*_*p*_^2^ = 0.29, indicated reduced practice effects in especially *R*_1_ and *R*_5_. The RSI group × Key × Block, *F*(36,792) = 3.5, *p* < 0.001, *η*_*p*_^2^ = 0.14, showed that these element-specific learning effects were different in the two groups. The RSI group × Block interaction did not reach significance, *F*(6,132) = 1.1, *p* > 0.20, implying that improvement itself did not significantly differ for the two groups.

Planned comparisons of *T*_5_ with *T*_23467_ were carried out to determine whether the present sequences involved a spontaneously developing slow *R*_5_, just like in previous research. These showed that this relatively slow *R*_5_ occurred across all blocks in both groups, *F*s(1,22) > 8.3, *p*s < 0.01, *η*_*p*_^2^s > 0.27. In fact, in both RSI groups *T*_5_ was significantly different from *T*_23467_ already in Block 1, *F*s(1,22) > 4.7, *p*s < 0.05, *η*_*p*_^2^s > 0.18, and this difference remained significant in each successive practice block, *F*s(1,22) > 7.2, *p*s < 0.05, *η*_*p*_^2^s > 0.25 (Note from Fig. 1 that in Block 1 of the long-RSI group this difference was not yet caused by a relatively long *T*_5_).

An ANOVA with the above mentioned design was used to analyze arcsine transformed error proportions. It showed that error rate monotonously increased over the successive blocks, from 1.2% per key in Block 1 to 2.4% in Block 7, *F*(6,132) = 8.1, *p* < 0.001, *η*_*p*_^2^ = 0.27. The slow *R*_5_ appeared to also have the highest error rate: error rates of *R*_123467_ were all below 2.0% whereas *R*_5_ had an average error rate of 3.7%, *F*(6,132) = 13.9, *p* < 0.001, *η*_*p*_^2^ = 0.39. As indicated by the Group × Key interaction, *F*(6,132) = 3.8, *p* < .05, *η*_*p*_^2^ = 0.15, this relatively high error rate at *R*_5_ differed somewhat in both RSI groups (0-RSI group, *R*_5_: 4.6% vs. *R*_123467_: 1.7%; long-RSI group, *R*_5_: 2.8% vs. *R*_123467_: 1.4%), but planned comparisons showed that in both RSI groups the error rate at *R*_5_ was higher than at *R*_23467_, *F*s(1,22) > 5.3, *p*s < 0.05, *η*_*p*_^2^s < 0.19.

### Test phase

The test phase response times were subjected to a 2 (RSI group) × 2 (RSI condition: 0-RSI vs. long-RSI) × 2 (Familiarity: familiar vs. unfamiliar sequence) × 7 (Key) mixed ANOVA with RSI group as between-subjects variable. All four main effects were significant: RSI group, *F*(1,22) = 5.7, *p* < 0.05, *η*_*p*_^2^ = 0.21, RSI condition, *F*(1,22) = 17.8, *p* < 0.001, *η*_*p*_^2^ = 0.45, Familiarity, *F*(1,22) = 9,9, *p* < 0.01, *η*_*p*_^2^ = 0.31, and Key, *F*(6,132) = 12.9, *p* < 0.001, *η*_*p*_^2^ = 0.37. These main effects showed that RTs were shortest in the 0-RSI group (408 vs. 520 ms), mean RTs were shorter in the 0-RSI than in the long-RSI ms test condition (421 vs. 507 ms), and familiar sequences were executed faster than unfamiliar sequences (450 vs. 478 ms).

**Fig. 2:**
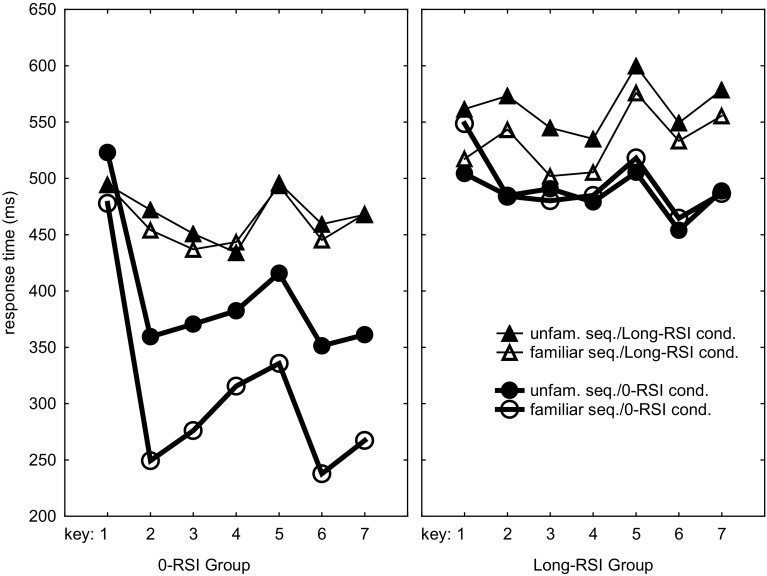
Response times in the test phase as a function of sequence familiarity and RSI condition in the 0-RSI and long-RSI groups

The most important result is depicted in Fig. 2 and this result is corroborated by a significant RSI group × RSI condition × Familiarity interaction, *F*(1,22) = 11.1, *p* < 0.01, *η*_*p*_^2^ = 0.34. This interaction provides direct support for the expectation that the difference between familiar and unfamiliar sequences of each RSI group is largest for the RSI condition the participants had been practicing in. Planned comparisons confirmed this in that the advantage of the familiar over the unfamiliar sequence was significant for the 0-RSI group in the 0-RSI condition, *F*(1,22) = 15.7, *p* < 0.001, *η*_*p*_^2^ = 0.42 (308 vs. 395 = 87 ms, see left frame of Fig. 2) while this group did not execute the familiar sequence faster in the long-RSI condition, *F*(1,22) = 0.2, *p* = 0.68 (462 vs. 467 = 5 ms). For the 0-RSI group, the familiarity effect was indeed significantly different in the two RSI conditions, *F*(1,11) = 8.9, *p* = 0.01, *η*_*p*_^2^ = 0.45. Conversely, for the long-RSI group the advantage of the familiar over the unfamiliar sequence was significant in the long-RSI condition, *F*(1,22) = 5.2, *p* = .03, *η*_*p*_^2^ = 0.19 (533 vs. 563 = 30 ms), and not so in the 0-RSI condition, *F*(1,22) = 0.2, *p* = 0.69 (495 vs. 487 = − 8 ms). This time, however, the familiarity effect was not statistically different in the two RSI conditions, *F*(1,11) = 2.7, *p* = 0.12. Together, these results support the notion that practice with a 0 RSI and with long RSIs yield different representations that cannot be used well in the other RSI condition—though the effect was weaker in the long-RSI group.

To test whether each RSI group had developed a sequencing skill that they could use with unfamiliar sequences too, we examined whether each RSI group was faster reproducing the unfamiliar sequences in the RSI condition they had practiced with. This showed that the 0-RSI group was indeed faster than the long-RSI group executing unfamiliar sequences in the 0-RSI condition, *F*(1,22) = 4.2, *p* = 0.05, *η*_*p*_^2^ = 0.16 (Fig. 2). Such an advantage for the long-RSI group in the long-RSI condition was not significant, *F*(1,22) = 2.7, *p* = 0.11, and in fact the long-RSI group executed the unfamiliar sequence non-significantly slower, instead of faster, than the 0-RSI group. This indicates that responding to individual stimuli is a skill that the 0-RSI group possessed as well as the long-RSI group.

Like in the practice phase, *R*_5_ appeared to be slower than *R*_23467_ in each of the 8 test conditions (see Fig. 2), *F*s(1,22) > 20.2, *p*s < 0.002, *η*_*p*_^2^s > 0.48. We further found a RSI Group × RSI condition × Key interaction, *F*(6,132) = 3.5, *p* < 0.01, *η*_*p*_^2^ = 0.14, that seems to have been caused by *R*_1_ being relatively slow for both unfamiliar and familiar sequences in the 0-RSI condition for the 0-RSI group. Most likely, this effect was sufficiently strong to also yield the significant RSI group × Key, *F*(6,132) = 4.8, *p* < 0.001, *η*_*p*_^2^ = 0.18, and RSI condition × Key interactions, *F*(6,132) = 18.0, *p* < 0.001, *η*_*p*_^2^ = 0.45, that we observed.

The above ANOVA design was used to analyze the arcsine transformed error proportions in the test phase too. This ANOVA showed that the 0-RSI group generally made more errors than the long-RSI group (2.8 vs. 1.9% per key), *F*(1,22) = 4.5, *p* < .05, *η*_*p*_^2^ = 0.17. The significant Key main effect indicated that *R*_5_ had again more errors than the other keys (*R*_5_: 4.7% vs. *R*_123467_: below 2.7%), *F*(6,132) = 12.5, *p* < 0.001, *η*_*p*_^2^ = 0.36, which was confirmed by a *R*_5_ vs. *R*_23467_ planned comparison, *F*(1,22) = 38.3, *p* < .001, *η*_*p*_^2^ = 0.64. This relatively high error rate in *R*_5_ appeared more pronounced in the 0-RSI condition (*R*_5_ vs. *R*_123467_: 5.2 vs. 1.8%) than in the long-RSI condition (*R*_5_ vs. *R*_123467_: 4.2 vs. 2.1%), *F*(6,132) = 3.1, *p* < 0.01, *η*_*p*_^2^ = 0.12.

### Awareness task

The result of the awareness task showed that of the 12 participants in the 0-RSI group, 2 participants reproduced their 2 sequences perfectly in both the spatial and the verbal tests (i.e., 17% of the total of 24 reproduced sequences). In the long-RSI group, only 1 participant reproduced in the spatial test 1 of the 2 sequences without error (i.e., 4% of all sequences).

We performed a nonparametric mixed 2 (RSI group) × 2 (Task) × 7 (Key) ANOVA with RSI group as between-subjects variable on the numbers of correct responses per sequential position using the F1-LD-F2 design (of the nparLD package, Noguchi, Gel, Brunner, & Konietschke, 2012) in R Studio (version 1.0.136, R Core Team, 2013). This analysis showed that the number of correct responses was higher in the 0-RSI (54%) than in the long-RSI group (37%), WTS(1) = 5.9, *p* = 0.02^2^. It further showed that the number of correct responses did not differ significantly between the spatial and verbal tests, WTS(1) = 1.9, *p* = 0.17, and neither was this the case across the various key positions, WTS(6) = 8.1, *p* = 0.22. However, the Task × Key interaction, WTS(6) = 19.8, *p* = 0.003, indicated that in the verbal test error rates were higher at *R*_1_, *R*_2_ and perhaps *R*_7_, while in the spatial test they were higher for *R*_5_ and *R*_6_. This difference suggests that verbal and spatial sequence reproduction involved different reproduction strategy.

We repeated this analysis on only the faster responses to reduce possible contamination by reconstruction of element order on the basis of other sequence knowledge. We, therefore, arbitrarily counted only responses that were given faster than the averages per key across all participants in the verbal and spatial tests. These amounted to 2253, 1110, 933, 942, 951, 892, and 908 ms for *R*_1_–*R*_7_, respectively^3^. This analysis showed an average of 22% fast, correct responses. There was no longer a difference between 0-RSI and long-RSI participants, WTS(1) = 0.7, *p* = 0.41. Instead, there were now more correct responses in the spatial than in the verbal test (27 vs. 16%), WTS(1) = 7.6, *p* = 0.006, suggesting that spatial sequence knowledge was more rapidly available than verbal sequence knowledge.

## Discussion

The present results provided support for the hypothesis that skill in discrete keying sequences can be used only at the execution rate at which the task had originally been practiced. We established this in an experiment in which two groups practiced the same sequences with RSIs of either 0, or varying between 500 and 2000 ms. The ensuing test phase involved conditions with familiar and unfamiliar sequences under the same and under different RSI conditions as the participants had been practicing in. We further examined with a new, computer-based awareness task whether sequence knowledge in the DSP task is perhaps more explicit when RSIs are longer, but the results did not support this idea.

### RSI-specific skills

The 0-RSI participants showed an execution rate advantage of familiar over unfamiliar sequences in the 0-RSI test condition and not in the long-RSI test condition. Given that the present 0-RSI practice phase involved the type of practice assumed to yield motor chunks, we conclude that motor chunks cannot be used at low sequence execution rates. The fact that in the long-RSI condition these 0-RSI participants did not carry out familiar sequences faster than unfamiliar sequences shows that at low execution rates 0-RSI participants again executed familiar sequences in the reaction mode. No indications were observed that suggest that in the 0-RSI participants the associative mode had facilitated sequences with the long RSIs. This is reasonable given that longer RSIs are associated with a decay of activity (Soetens et al., 2004).

These findings correspond to a similar lack of transfer found earlier when DSP sequences had been practiced with 0 RSIs, and were then hidden in an SRT task with its 200 ms RSIs (Verwey, 2003b). In that study, the lack of transfer was attributed to the participants being unaware that familiar DSP sequences were part of the SRT task. In the present study, however, participants had been explicitly informed that they were executing the familiar sequences at another rate. So, in line with the earlier conclusion that the two tasks rely on different types of knowledge (Verwey & Abrahamse, 2012; Verwey & Wright, 2014), the earlier lack of transfer between the tasks (Verwey, 2003b) can be attributed to the reliance on different representations in the DSP and SRT tasks.

The long-RSI participants showed statistically significant faster execution of familiar than of unfamiliar sequences in the long-RSI test condition, and not in the 0-RSI condition. However, as indicated by non-significance of the familiarity by RSI condition interaction, RSI-specific learning was less pronounced for this group than for the 0-RSI group. These results suggest that long-RSI participants developed sequence knowledge that cannot be used when RSI is 0. Given the limited explicit knowledge in the long-RSI participants, and the similarity of the long-RSI task and the SRT task, we believe that improvement of these participants in the DSP task relies on the associative learning found with SRT tasks too (Abrahamse et al., 2010; Keele et al., 2003). This is not unexpected as earlier DSP task studies showed that associative learning develops in normal DSP sequences too, both in younger participants (Barnhoorn et al., 2016; Ruitenberg, Verwey, Schutter, & Abrahamse, 2014; Verwey & Abrahamse, 2012), and in older participants (Barnhoorn et al., 2016; Verwey, 2010; Verwey et al., 2011). It is remarkable, though, that long-RSI participants did not seem to have benefitted from associative sequence knowledge after RSI had changed. This may imply that the associative mode is RSI-specific too. In any case, the present results do not confirm that the long-RSI participants relied for sequence execution on explicit sequence knowledge. Most likely, the long-RSI participants had not spontaneously tested hypotheses on the order of the sequence elements, like many participants in the SRT task seem to do (Frensch & Miner, 1994; Rünger & Frensch, 2008).

The data further demonstrate that in the 0-RSI test condition even unfamiliar sequences were executed more rapidly by 0-RSI participants than by long-RSI participants. Practicing discrete keying sequences with 0-RSIs not only appears to induce learning of a particular sequence, but also some general, sequence-unspecific skill to produce discrete keying sequences. This skill, then, involves the proficiency to first activate several responses in memory that are then executed in a single burst. We earlier modelled this with C-SMB by assuming that a cognitive processor loads responses into a motor buffer in a cognitive loop from which the elements are then read and executed by the motor processor in a motor loop (Verwey et al., 2015; also see Fendrich et al., 1991; Sternberg, Monsell, Knoll, & Wright, 1978; Verwey, 1996). This general sequencing skill might involve an increase in dexterity to press successive keys (Parikh & Cole, 2014). This distinction between a sequence-specific and a general sequencing skill corresponds with earlier indications in the DSP task that middle-aged and elderly participants showed similar improvement in terms of absolute execution times, but that in elderly participants there was more sequence-unspecific learning (Verwey, 2010; Verwey et al., 2011). The present results now indicate that younger adults show sequence-unspecific learning too.

It is reasonable that no indications were observed for a general keying skill in the long-RSI group as executing unfamiliar sequences with long RSIs involves a mere reacting to stimuli in the reaction mode. This is something that 0-RSI participants had experience with in early practice, too, but in fact, it is quite unlikely that any participant would need much practice to learn this.

### Explicit sequence knowledge

The new computerized awareness task we introduced was designed to distinguish explicit verbal and spatial sequence knowledge while also reducing contamination by implicit sequence knowledge. This awareness task showed considerably lower awareness than the previously used paper-and-pencil tests, even when the slower responses were not removed: While across 6 DSP studies with 6- and 7-element, 0-RSI sequences, 53% of the participants had been able to write down both their sequences (Verwey et al., 2016), in the present task only 17% of the 0-RSI participants showed full explicit sequence knowledge. This low awareness level occurred even though in the present spatial test the visual display was identical to that in the actual DSP keying task. That the present awareness task showed much lower levels of explicit sequence knowledge than the traditional paper-and-pencil tests may have been caused by the use of another response modality, pointing with the mouse instead of writing. In addition, the present participants may have experienced some time pressure because the experimenter remained in the room during the computerized awareness task.

Theoretically, the so-called single-system view assumes that implicit and explicit knowledge are rooted in the same system and differ merely in strength (Cleeremans & Jiménez, 2002; Shanks & Perruchet, 2002). In contrast, the multiple-system view implies that explicit and implicit learning are supported by different memory systems (Reber & Squire, 1998). We propose that the many indications for different types of implicit sequence knowledge in the DSP task are consistent with a multiple systems view in the sense that different representational systems are responsible for different types of sequence knowledge. In line with the single-system view, however, knowledge may become explicit when the knowledge in each of these systems is sufficiently strong.

### Concatenating segments

Earlier studies with 7-key DSP sequences showed a relatively slow *R*_5_ (De Kleine & Verwey, 2009; Ruitenberg, De Kleine, Van der Lubbe, Verwey, & Abrahamse, 2012; Verwey, Abrahamse, De Kleine, & Ruitenberg, 2014). This spontaneously developing slow response was attributed to the concatenation of successive motor chunks because motor chunks would be limited to 3–5 responses (e.g., Abrahamse et al., 2013; Verwey, 2003a; Verwey & Eikelboom, 2003). The present finding of a slow *R*_5_ shows that this slow response is not specific for the sequences used in those earlier studies, as we here used different sequences^4^.

The present finding that in the DSP task segmentation occurs already early in practice has been observed before (De Kleine & Verwey, 2009; Ruitenberg, De Kleine, et al., 2012; Verwey et al., 2014). This rapid development of segmentation in several studies suggests that the slow response halfway through a sequence may be caused by the limited capacity of working memory, which has a capacity of about four elements too (Cowan, 2000). That is, segmentation may be attributed to a capacity limitation of central-symbolic sequence representations rather than of motor chunks^5^. With more extensive practice than the typical 500 trials per sequence in DSP studies motor chunks may, therefore, gradually grow longer than the typical 3–5 elements (as suggested also by data reported by Acuna et al., 2014).

## Conclusions

The present results confirm the hypothesis that practicing DSP keying sequences induces different representations for practice with 0 RSIs and with longer RSIs, and that these representations can be used only with the same RSIs as used during practice. According to our computerized sequence awareness task the sequence knowledge that developed with long-RSI practice was not explicit, and it may, therefore, consist of associative sequence knowledge. In addition, practicing short, discrete sequences with 0-RSIs seems to yield the general skill of preparing and then executing several movements, irrespective of their order. The observation of a slow *R*_5_ in familiar and unfamiliar sequences in both RSI groups may well have been caused by a limited working memory capacity instead of by a motor chunk limitation. With extensive practice motor chunks may, therefore, contain more than 3–5 key presses.

## Footnotes

1 You may realize this when you type your PIN on the keypad of a computer keyboard rather than on an ATM.

2 Effect sizes have not yet been developed for this type of ANOVA.

3 This is still clearly longer than RTs obtained during sequence execution (see Fig. 2). We initially considered using the mean RTs measured during execution of the sequences as threshold. However, the awareness task also included moving the mouse and clicking the mouse button which obviously takes longer than pressing a key on which a finger is already resting during sequence executing.

4 Interestingly, in a data entry task with 7-digit sequences that involved no practice, it was *R*_4_ that was relatively slow (Fendrich and Arengo 2004).

5 Recent results indicate that in older adults this slow response may also be attributed to a slow finger (Barnhoorn et al. 2017).
